# Suicide genes: monitoring cells in patients with a safety switch

**DOI:** 10.3389/fphar.2014.00241

**Published:** 2014-11-06

**Authors:** Linda G. Eissenberg, Michael Rettig, Farrokh Dehdashti, David Piwnica-Worms, John F. DiPersio

**Affiliations:** ^1^Department of Internal Medicine, Washington University School of Medicine, St. LouisMO, USA; ^2^Department of Radiology, Washington University School of Medicine, St. LouisMO, USA; ^3^Department of Cancer Systems Imaging, University of Texas MD Anderson Cancer CenterHouston, TX, USA

**Keywords:** suicide gene, gene therapy, thymidine kinase, PET-imaging, transplant biology, regenerative medicine

## Abstract

Clinical trials increasingly incorporate suicide genes either as direct lytic agents for tumors or as safety switches in therapies based on genetically modified cells. Suicide genes can also be used as non-invasive reporters to monitor the biological consequences of administering genetically modified cells to patients and gather information relevant to patient safety. These genes can monitor therapeutic outcomes addressable by early clinical intervention. As an example, our recent clinical trial used ^18^F-9-(4-fluoro-3-hydroxymethylbutyl)guanine (^18^FHBG) and positron emission tomography (PET)/CT scans to follow T cells transduced with herpes simplex virus thymidine kinase after administration to patients. Guided by preclinical data we ultimately hope to discern whether a particular pattern of transduced T cell migration within patients reflects early development of graft vs. host disease. Current difficulties in terms of choice of suicide gene, biodistribution of radiolabeled tracers in humans vs. animal models, and threshold levels of genetically modified cells needed for detection by PET/CT are discussed. As alternative suicide genes are developed, additional radiolabel probes suitable for imaging in patients should be considered.

## CLINICAL SAFETY SWITCHES

Suicide genes convert innocuous prodrugs into cytotoxic products. Injecting viruses carrying suicide genes directly into solid tumors destroys the virus-infected cells and nearby neighbors through diffusion of cytotoxic products. Inserting suicide genes into cells that migrate to tumors [autologous tumor cells, mesenchymal stem cells, neural stem cells, and immune cells such as T cells, NK cells, and genetically modified chimeric antigen receptor T (CAR-T) cells] can accomplish the same task.

Leukemia patients are often administered allogeneic T cells to attack their cancerous cells. Unfortunately T cell replete transplants with the most robust anti-tumor activity tend to have the greatest potential to cause graft vs. host disease (GvHD), a frequent and sometimes fatal side effect of allogeneic stem cell transplantation. GvHD manifests itself early after transplantation and after the overt elimination of leukemia. T cells genetically modified to express the herpes simplex virus thymidine kinase (TK) suicide gene can be killed by administering the prodrug ganciclovir (GCV) to induce apoptosis at the first sign of GvHD (Mercier-Letondal et al., 2008; Zhan et al., 2013). Although CAR-T cells provide a more targeted type of T cell mediated anti-tumor therapy, the potential for GvHD or some off-target tissue damage is not eliminated and the need for a suicide gene safety switch cannot be underestimated.

Safety switches (suicide genes) are of particular value in therapies dependent upon long-lived and/or proliferating cells. Genetically modified cells carry an inherent and potentially life-long hazard of cancerous transformations. Stem cells administered to regenerate tissues damaged by disease or treatment, correct congenital malformations, or rejuvenate aging tissues may have unknown risks (Mavroudi et al., 2014). Likewise there could be unintended consequences from administering autologous cells modified *ex vivo* to act as in-patient factories to produce biological molecules, such as insulin, to alleviate the need for repeated injections (Sanlioglu et al., 2012).

## REPORTERS TO MONITOR CLINICAL SAFETY AND BIOLOGY

Monitoring the location of genetically modified cells in patients could have important ramifications in terms of either safety or the general biology of these therapeutic interventions. Do the cells reach their target or migrate to an unexpected location, cause damage to a normal tissue, or contribute to tumor progression? If a cancer relapses, do modified immune cells reactivate, proliferate, and target the new tumor or ignore it? Is an organ regenerated primarily from genetically modified stem cells recruited to enhance tissue repair? What is the relative amount of modified cells at various sites? Do *ex vivo* manipulations of transduced cells affect their therapeutic potential and biodistribution? The limited penetration of visible light through human and large animal bodies makes answering these questions by tagging the cells with bioluminescent reporter probes (luciferase) untenable. Also, there are no Food and Drug Administration (FDA) approved reagents for generation and measurement of bioluminescent imaging in humans. In these situations higher energy reporters are needed, such as those suitable for imaging via positron emission tomography (PET) scans of patients.

## HERPES SIMPLEX VIRUS THYMIDINE KINASE

All enzyme-based suicide genes should amplify tracer signals, but to be a successful reporter the product of the reaction must also be retained in the cells. Conversion of 5-fluorocytosine by cytosine deaminase to a molecule that diffuses out of the cell, 5-fluorouracil, makes it a poor reporter. In contrast TK phosphorylates cellular substrates (ganciclovir, penciclovir, and derivatives) into membrane impermeable phosphorylated forms and entraps them within cells. This makes TK a better choice for imaging studies.

Following administration of a cationic liposomal vector carrying the suicide gene in an expression plasmid, TK was first monitored in five glioblastoma patients using the substrate ^124^I-labeled 2′-fluoro-2′-deoxy-1β-D-arabino-furanosyl-5-iodo-uracil (^124^I-FIAU; Jacobs et al., 2001). ^124^I-FIAU-PET imaging detected a brain tumor in only one of the patients. Since histology indicated that there were significantly fewer cells in the tumors of the remaining four patients, the authors theorized that a threshold number of labeled cells must be present before ^124^I-FIAU-PET scans can detect them.

A later study used 9-[4-[^18^F]fluoro-3-(hydroxymethyl)butyl]guanine (^18^F-FHBG) as a substrate for TK (Penuelas et al., 2005). The enzyme was carried by an adenovirus injected directly into liver tumors. The tumors were visible by ^18^F-FHBG–PET in the initial scan before a 2-week valganciclovir treatment was begun only in patients receiving a threshold of ≥10^12^ virus particles, and disappeared by day 7 of treatment.

In 2009 cells were first modified *ex vivo* to carry a suicide gene as both a safety switch and a reporter in patients (Yaghoubi et al., 2009). An IL-13 zetakine gene was included to improve tumor targeting by autologous CD8+ T cells, and a single patient was evaluated. ^18^F-FHBG–PET imaging successfully localized the T cells to both a resected glioma and an adjacent tumor that had formed after the resection.

Our own recently completed trial, to be reported in detail elsewhere (Infusion of genetically modified T cells: a pilot study of tracking and toxicity, IND 11917, clinicaltrials.gov #NCT00871702), is the only additional clinical study of which we are aware that uses ^18^F-FHBG–PET to follow TK-modified cells. Allogeneic donor T cells were retrovirally transduced to carry a TK-based suicide gene both as a means of controlling GvHD and to facilitate tracking of their migration in patients undergoing a donor lymphocyte infusion (DLI). To achieve both greater sensitivity to GCV and more accumulation of the phosphorylated ^18^F-FHBG for imaging, we used, TK75, a hyperactive mutated version of the enzyme (Black et al., 1996). TK75 was fused to the extracellular and transmembrane domains of the human CD34 protein (CD34-TK75) to facilitate selection of transduced cells using a Miltenyi CliniMACS CD34 reagent system during manufacturing and detection of the cells by flow cytometry both during production and in the peripheral blood of patients.

Our preclinical bioluminescence studies suggested that allogeneic T cells migrate in a distinctive pattern when they cause GvHD in mice (Nervi et al., 2007). Finding such a pattern in patients could provide both a non-invasive means to track these cells in human DLI recipients and a surrogate marker to predict which patients are most likely to develop GvHD. In our phase I study the first two patients who received purified CD34-TK75-transduced cells as a DLI had no correlative ^18^F-FHBG-PET/CT imaging. The remaining six individuals were scanned at three time points. Because in a DLI setting acute GvHD is usually not evident before 30 days post administration of T cells, we chose to do one baseline scan before the DLI, and additional scans on ∼days +14 and +30 after the infusion of the CD34-TK75 genetically modified DLI.

**FIGURE 1:**
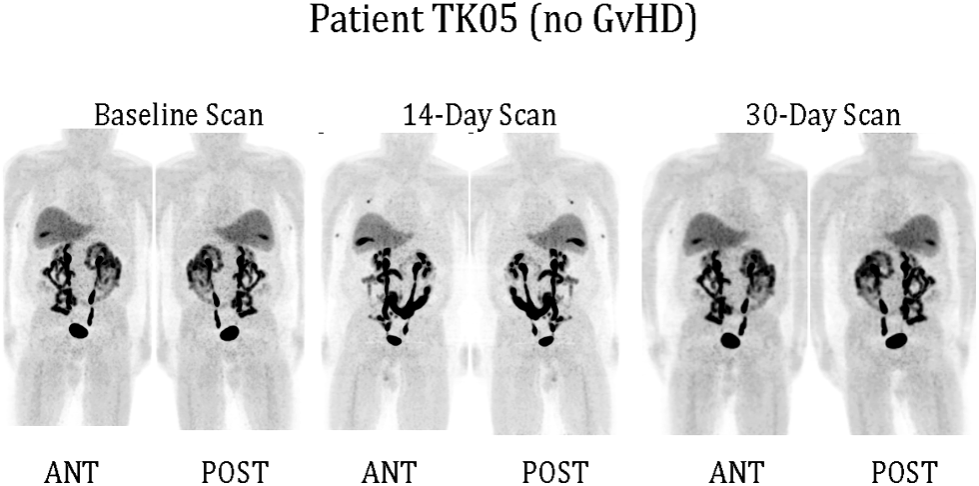
**^**18**^F-FHBG-PET/CT scans of patients.** Under FDA IND #11917 (clinicaltrials.gov #NCT00871702) patients received 0.1–1.3 × 10^6^ purified allogeneic CD34-TK75-transduced cells/kg as a donor lymphocyte infusion (DLI). ^18^F-FHBG-PET/CT scans were performed at baseline before DLI, and on ∼days +14 and +30 after DLI. Anterior and posterior reprojection images of patient TK05 are shown. Scans were made 60 min after administration of ^18^F-FHBG and show a normal distribution of the radiotracer. No reproducible difference was detected between the baseline and later scans for any patient. TK05 did not develop graft vs. host disease (GvHD).

**FIGURE 2:**
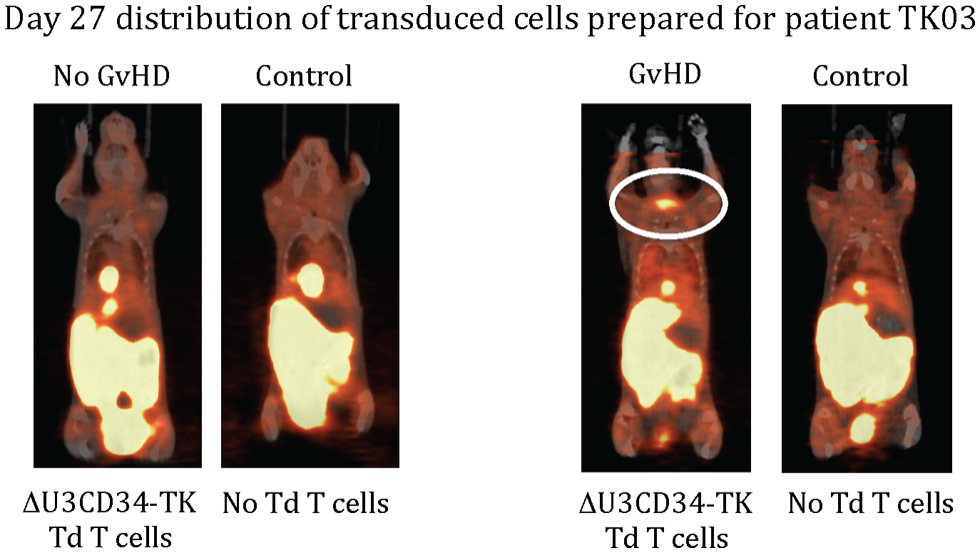
**^**18**^F-FHBG-PET/CT scans of mice.** Two NSG mice were injected retro-orbitally with 2 × 10^6^ of the same CD34-TK75 cells provided to each patient. On days +7 and +27 each of these mice was paired with an untreated mouse and they were simultaneously injected with the same preparation of ^18^F-FHBG that the patients received, then imaged 1 h later using ^18^F-FHBG microPET/CT. At this dose about half the mice get GvHD, and only those mice had concentrated radiotracer in the thymic region (circled).

Only one of our six imaged patients developed GvHD (day 64) potentially attributable to the genetically modified T cells. Unfortunately no discernable difference was observed between the biodistribution of ^18^F-FHBG in this or any other patient at baseline and later time points (**Figure 1**). We used a portion of the same CD34-TK75 transduced donor T cells and ^18^F-FHBG prepared for the patients under GMP conditions in a parallel transplant of 2 NOD-SCIDg-/-(NSG) mice for each patient. As expected the xenogeneic CD34-TK75 transduced T cells induced GvHD in NSG mice, and the ^18^F-FHBG-microPET/CT scans revealed transduced T cells in their thymic region (**Figure 2**). We previously demonstrated by BLI that this site is one of the preferred locations for migration and expansion of human T cells during xenogeneic GvHD (Nervi et al., 2007). Our current data recapitualted this distinctive migration pattern and also reflects the feasibility of in-house cGMP production of both functional transduced suicide gene expressing human T cells and ^18^F-FHBG.

Data from other trials suggested that the therapeutic potential of T cells is affected by the means in which T cells are activated pre-transduction and the method of expansion thereafter (review Cieri et al., 2014). Our cells were activated with IL2 and anti-CD3/anti-CD28 coated beads, and expanded for only 2–3 days prior to infusion into patients. While the cells functioned as expected in our xenograft model and persisted for at least 6 months (latest time point evaluated), it is possible that their function was sub-optimal in patients. Including HSV-TK as a reporter in future studies will provide another metric to correlate therapeutic outcome with *ex vivo* manipulations prior to infusion.

We noted that background ^18^F-FHBG accumulation was negligible throughout the body in all human DLI recipients. In contrast, it was difficult or impossible to track CD34-TK75 expressing human T cells trafficking to the abdomen of mice due to the high background accumulation of ^18^F-FHBG in the abdomen. As discussed earlier, a threshold level of radiotracer may be required before it can be detected by imaging. Thus it is likely that due to the very limited number of transduced T cells available for infusion (0.1–1.3 × 10^6^ CD3/kg), we failed to reach that threshold in any patient. Alternatively a diffuse distribution of the transduced cells, an inappropriate choice of time points, or a combination of these factors may have limited our ability to detect an alteration in the pattern of uptake of ^18^F-FHBG in the tissues of human recipients after DLI. In collaboration with MolMed we have initiated a second trial of ^18^F-FHBG-PET/CT imaging (IND14367, clinicaltrials.gov #NCT00914628) in which patients that have poor immune recovery after haploidentical transplant will be infused with TK transduced T cells from the same haploidentical donor between 40 and 60 days post transplant. In this trial ≥50 times as many TK transduced cells/kg will be administered.

A concern for long-term cell-based gene therapies is that genetically modified cells might be immunologically recognized and eliminated. Although patients in our trial exhibited neither a cellular nor serum immune response to CD34-TK75, an immune response against unmodified TK has been noted in other trials (reviews Berger et al., 2006; Traversari et al., 2007). To address this concern investigators have developed mutated or truncated versions of human mitochondrial TK 2 that should be less immunogenic (Ponomarev et al., 2007; Campbell et al., 2012), although they have not yet been tested in patients. GCV used to treat cytomegalovirus infections, common after allogeneic stem cell transplantation, can also eliminate TK-modified cells, so other forms of CMV treatment and prophylaxis need to be considered if TK is the suicide gene chosen. GCV only kills TK-bearing cells that are actively proliferating, so it is theoretically possible that administration of the drug might fail to eliminate all genetically modified cells. This might be advantageous in the allogeneic transplant setting where the most proliferative alloreactive T cells would be preferentially eliminated leaving T cells reactive to other antigens and the leukemia intact, thus enhancing the chances for immune reconstitution and a robust graft vs. leukemia effect.

The biodistribution of 2 radioactive substrates used with TK, ^18^F-FHBG and 2′-deoxy-2′-[^18^F]fluoro-5-methyl-1-β-D-arabinofuranosyluracil (^18^F-FMAU), has been compared in healthy volunteers (Campbell et al., 2012). More ^18^F-FMAU than ^18^F-FHBG accumulated in the liver, bladder, and myocardium while more ^18^F-FHBG than ^18^F-FMAU was concentrated in the gall bladder and gastrointestinal tract. This observation indicates that one TK substrate for PET imaging may be preferable over another depending upon the anticipated location of the genetically modified cells. This study also demonstrated that preclinical models may not accurately reflect the distribution of the substrates in humans since overall there was more background in mice when using ^18^FHBG than ^18^F-FMAU, particularly in the gastrointestinal tract, bladder, and spleen. In comparison, there was less overall accumulation of ^18^F-FHBG in patients than in mice, just as in the study we report here. The reverse was true for ^18^F-FMAU.

There are currently a number of suicide genes under preclinical investigation. Developing additional highly retained radiolabeled probes for imaging that have high signal to noise ratios could be of significant value to patients and the general biological understanding in this field.

## PERSPECTIVE

Suicide genes such as TK should be considered an adjunct to any clinical gene therapy trial in order to exploit their dual safety and monitoring functions. Many factors govern which suicide gene system is optimal. Among these are the anticipated urgency to rid a patient of the cells, whether it is better to be able to leave non-proliferating genetically modified cells intact or to kill all transduced cells, the overall potency of a particular system, the importance of bystander-cell killing, and immunogenicity. The potential to gather safety or general biological information by directly imaging the genetically modified cells in the patient should be added to these considerations.

There are hurdles for performing any gene therapy trial. Adding a component that requires synthesis of a radioactive probe admittedly introduces additional complexities, but these are not insurmountable. FDA and institutional oversight was required for the cGMP quality manufacturing of both our genetically modified cells and ^18^F-FHBG, each of which required specialized facilities, highly trained individuals, and separate clinical protocols. Nonetheless, because preclinical models do not always reflect the biology of either healthy humans or patients, there is a marked benefit to including a tool to gather additional biologic information in a non-invasive manner. As transplant biology and regenerative stem cell therapy advance, there is an increasing need to monitor and understand the behavior of various exogenously added cells in patients.

## AUTHOR CONTRIBUTIONS

All authors contributed to the conception, design, acquisition, analysis, or interpretation of the work. All authors also drafted the work or critically evaluated it for intellectual content, approved the final version, and agree to be accountable for all aspects of the work.

## Conflict of Interest Statement

The authors declare that the research was conducted in the absence of any commercial or financial relationships that could be construed as a potential conflict of interest.
